# The Role of Pharmacists in Preventing Falls among America’s Older Adults

**DOI:** 10.3389/fpubh.2016.00250

**Published:** 2016-11-09

**Authors:** Mamta V. Karani, Yara Haddad, Robin Lee

**Affiliations:** ^1^Division of Unintentional Injury Prevention, National Center for Injury Prevention and Control, Centers for Disease Control and Prevention, Atlanta, GA, USA

**Keywords:** accidental falls, older adult, polypharmacy, pharmacists, medication therapy management, STEADI

## Abstract

Falls are the leading cause of both fatal and non-fatal injuries in people aged 65 years and older and can lead to significant costs, injuries, functional decline, and reduced quality of life. While certain medications are known to increase fall risk, medication use is a modifiable risk factor. Pharmacists have specialized training in medication management and can play an important role in fall prevention. Working in a patient-centered team-based approach, pharmacists can collaborate with the primary care providers to reduce fall risk. They can screen for fall risk, review and optimize medication therapy, recommend vitamin D, and educate patients and caregivers about ways to prevent falls. To help health-care providers implement fall prevention, the Centers for Disease Control and Prevention developed the Stopping Elderly Accidents, Deaths, and Injuries (STEADI) initiative. Based on the established clinical guidelines, STEADI provides members of the health-care team, including pharmacists, with the tools and resources they need to manage their older patients’ fall risk. These tools are being adapted to specifically advance the roles of pharmacists in reviewing medications, identifying those that increase fall risk, and communicating those risks with patients’ primary care providers. Through a multidisciplinary approach, pharmacists along with other members of the health-care team can better meet the needs of America’s growing older adult population and reduce falls.

## The Issue

Falls are the leading cause of both fatal and non-fatal injuries in people aged 65 years and older and can lead to significant costs, injuries, functional decline, and reduction in the quality of life (1). In 2014, over 27,000 older adults died from a fall and 2.8 million more required treatment in emergency departments for non-fatal injuries (1). Nevertheless, falling is not a normal part of aging and can be prevented.

Falls can be attributed to a number of modifiable risk factors including gait and balance problems, vitamin D deficiency, vision impairment, foot ailments, and medication use (2–4). Polypharmacy, known as the use of multiple medications or the administration of more medications than clinically indicated, is common in older adults (5). Approximately 85% of older adults take at least one prescription medication and about 25% take five or more (6). Medications that affect the central nervous system can cause side effects that increase the chances of falling, such as dizziness, sedation, confusion, blurred vision, and orthostatic hypotension (7–10). Medication classes strongly associated with falls include anticonvulsants, antidepressants, antipsychotics, benzodiazepines, opioids, and sedative hypnotics (11). Benzodiazepines, including alprazolam and clonazepam, are prescribed in about 8% of older adults (12). During 2005–2010, roughly 5–7% of adults aged 60 years and older reported using hypnotics and sleep aids in the past 30 days (13). Additionally, when taken together, some of the aforementioned medications can have a synergistic effect on cognition and physical function, leading to a more pronounced fall risk (5, 14–17). Therefore, reviewing medications to see if medications can be stopped, switched, or reduced and managing those that may be clinically necessary are a key component to preventing older adult falls.

Many resources exist for use by health-care providers to optimize their patient’s medications and minimize polypharmacy and adverse events. Examples of resources include the Medication Appropriate Index (18), Beers criteria (11), Screening Tool of Older Person’s Prescriptions (STOPP)/Screening Tool to Alert doctors to Right Treatment (START) (19, 20), and the Anticholinergic Burden Index (21). While these tools exist, no one tool is all inclusive or considered the gold standard. Additionally, there is inconsistency among use of the tools. Therefore, each of these tools are helpful only when coupled with a thorough medication review by the primary care provider or if possible, a pharmacist.

Pharmacists are highly skilled in understanding how medications work individually or in combination to affect the body. Through a broad range of health-care services known as medication therapy management (MTM), pharmacists aim to ensure that each medication is the most effective and safest therapeutic option for a specific individual (22). Although MTM is a relatively new term, pharmacists have always been involved in medication review and management, despite their practice sites. A thorough medication review by a pharmacist includes a review of age-related physical changes that predispose older adults to drug–drug interactions, drug–disease interactions, and medication side effects that can increase the patient’s chances of falling (4, 23–25). For example, with age, the kidneys and liver may become less efficient, and the distribution of water and fat within the body changes. These physiological changes may affect the patient’s ability to metabolize medications, leading to exposure to higher doses, and an increased risk of adverse events. With every review, clinical pharmacists evaluate renal and hepatic functions to account for acute changes, modifying dose and/or frequency as needed (25, 26). In managing therapy, they consider health priorities and patient concerns, but always put patient safety and injury prevention as a priority.

To help health-care providers implement fall prevention, the Centers for Disease Control and Prevention (CDC) developed the Stopping Elderly Accidents, Deaths, and Injuries (STEADI) initiative. The initiative is specifically focused on reducing falls among community dwelling older adults and is based on the American and British Geriatrics Societies’ recommendations (27). STEADI provides members of the primary care team with the tools and resources they need to manage their older patients’ fall risk (28). The initiative encourages providers to take three initial steps to begin addressing their patients’ fall risks. They include (1) screening for fall risk by asking older adults if they have fallen in the past, feel unsteady, or are afraid of falling; (2) reviewing and managing their medications to determine if any increase fall risk and may need to be stopped, switched, or reduced; and (3) recommending vitamin D supplementation to improve bone, muscle, and nerve health.

While primary care physicians are trained on how to review and manage patients’ medications, research shows that physicians often lack a framework on how to do so and therefore are inconsistent when conducting medication reviews (29). Comprehensive medication reviews have also been mistaken for medication reconciliations, in which a medication list is updated by comparing the medical record to an external list of medications obtained from a patient, hospital, or other providers, but not necessarily evaluated for appropriateness (30). To assist primary care providers in conducting a comprehensive medication review, which includes a medication reconciliation in the process, CDC has developed a consistent approach called the Screen, Assess, Facilitate, and Educate (SAFE) method as part of the STEADI resources for providers (www.cdc.gov/STEADI). SAFE highlights four essential steps in conducting a medication review to reduce fall risk. Focusing on the patient and caregiver, this method is adapted from two reputable pharmacist practice tools, the pharmacist’s MTM and patient care processes (22, 31), and encourages collaboration with a pharmacist.

## Clinical Fall Prevention as a Team-Based Approach

Population growth, the aging population, and insurance expansions are projected to increase the demands for primary care physicians in the coming years (32). Due to time and resource constraints, the primary care physician’s ability to deliver preventive clinical services is often affected by the need to address acute illnesses, chronic illnesses, and patient requests, among other demands (33). While primary care physicians can be trained to perform fall risk assessments, comprehensive medication reviews may require 30–45 min to complete and may be challenging to perform in busy primary care settings (29). Clinical fall prevention efforts do not need to be the sole responsibility of primary care physicians. Research shows that a collaborative multidisciplinary team can provide individualized patient interventions and reduce the rate and risk of falls (34, 35).

Trained specifically in pharmacotherapy and medication management, pharmacists have been effective in regularly reviewing medications, managing health conditions, providing education, and delivering direct patient care (36, 37). Pharmacist-provided direct patient care has favorable effects across various patient outcomes, health-care settings, and disease states (38). Through MTM, pharmacists have successfully reduced the number of fall-related medications, provided clinically significant recommendations, and educated patients (and care providing team members) about medications and the risk of falls (39–41). By utilizing the expertise of additional health-care providers including nurses and pharmacists, the multidisciplinary team-based approach can alleviate some of the demands on primary care physicians while still ensuring optimal patient-centered care.

In addition to offering MTM, pharmacists are well positioned to aid the health-care team in conducting other fall prevention services (42). A pharmacist, in collaboration with the primary care provider, can screen patients using a standardized protocol to determine fall risk, complete a thorough medication review, and recommend vitamin D supplementation (when appropriate). When conducting a thorough review, the pharmacist can work with the patients and caregivers to *screen* for medications that may increase fall risk, *assess* the patient to best manage health conditions, *formulate* the patient’s medication action plan, and *educate* the patient and caregiver about medication changes and fall prevention strategies. If screening indicates a patient is at risk of falling, the pharmacist can coordinate with the primary care team to arrange a complete fall risk assessment. Understanding the various roles pharmacists may play, CDC is exploring options to gain a better understanding of how pharmacists in each setting can cost effectively provide fall prevention services.

## Future Steps

Through the STEADI initiative, health providers, community organizations, and state health departments have come together to care for our older adult population. The CDC is currently developing educational tools and resources to specifically advance the role pharmacists and other health-care providers can play in providing fall prevention services. Pharmacists may be motivated to engage in fall prevention for various reasons. While incentives can vary within each practice site, incentives include job satisfaction, patient safety, improved patient care, and financial incentives. Reimbursement varies depending on state laws and practice sites but may be possible through collaborative practice agreements, MTM billing, or structured payment models. Nevertheless, CDC is exploring options to better understand incentives, successes, and barriers to implementation of fall prevention. Conducting focus groups with pharmacists practicing in various sites, CDC is interested in understanding the falls related knowledge of pharmacists, learning potential barriers faced with providing fall prevention services, and developing a pharmacist-specific training on fall prevention services. CDC is also funding a project to learn best practices to improve collaboration and communication between community pharmacies and primary care offices. Through a multidisciplinary approach, pharmacists along with other members of the health-care team can better meet the needs of America’s growing older adult population and reduce falls.

## Author Contributions

All the authors made substantial contributions to conceptualizing the framework for the paper and in drafting the text. All the authors have reviewed the final document and agree with its content.

## Disclaimer

The findings and conclusions in this report are those of the authors and do not necessarily represent the official position of the Centers for Disease Control and Prevention.

## Conflict of Interest Statement

The authors declare that the research was conducted in the absence of any commercial or financial relationships that could be construed as a potential conflict of interest.

## References

[1] Bergen G Stevens MR Burns ER Falls and Fall Injuries Among Adults Aged ≥65 Years — United States, 2014. MMWR Morb Mortal Wkly Rep (2016) 65:993–8. 10.15585/mmwr.mm6537a2 27656914

[2] Ambrose AF Paul G Hausdorff JM. Risk factors for falls among older adults: a review of the literature. Maturitas (2013) 75(1):51–61. 10.1016/j.maturitas.2013.02.009 23523272

[3] Flicker L Mead K MacInnis RJ Nowson C Scherer S Stein MS Serum vitamin D and falls in older women in residential care in Australia. J Am Geriatr Soc (2003) 51(11):1533–8. 10.1046/j.1532-5415.2003.51510.x 14687381

[4] Huang AR Mallet L Rochefort CM Eguale T Buckeridge DL Tamblyn R. Medication-related falls in the elderly: causative factors and preventive strategies. Drugs Aging (2012) 29(5):359–76. 10.2165/11599460-000000000-00000 22550966

[5] Hajjar ER Cafiero AC Hanlon JT. Polypharmacy in elderly patients. Am J Geriatr Pharmacother (2007) 5(4):345–51. 10.1016/j.amjopharm.2007.12.002 18179993

[6] Kaufman DW Kelly JP Rosenberg L Anderson TE Mitchell AA. Recent patterns of medication use in the ambulatory adult population of the United States: the Slone survey. JAMA (2002) 287(3):337–44. 10.1001/jama.287.3.337 11790213

[7] Hartikainen S Lonnroos E Louhivuori K. Medication as a risk factor for falls: critical systematic review. J Gerontol A Biol Sci Med Sci (2007) 62(10):1172–81. 10.1093/gerona/62.10.1172 17921433

[8] Leipzig RM Cumming RG Tinetti ME. Drugs and falls in older people: a systematic review and meta-analysis: I. Psychotropic drugs. J Am Geriatr Soc (1999) 47(1):30–9. 10.1111/j.1532-5415.1999.tb01898.x 9920227

[9] Park H Satoh H Miki A Urushihara H Sawada Y. Medications associated with falls in older people: systematic review of publications from a recent 5-year period. Eur J Clin Pharmacol (2015) 71(12):1429–40. 10.1007/s00228-015-1955-3 26407688

[10] Woolcott JC Richardson KJ Wiens MO Patel B Marin J Khan KM Meta-analysis of the impact of 9 medication classes on falls in elderly persons. Arch Intern Med (2009) 169(21):1952–60. 10.1001/archinternmed.2009.357 19933955

[11] By the American Geriatrics Society 2015 Beers Criteria Update Expert Panel. American Geriatrics Society 2015 updated beers criteria for potentially inappropriate medication use in older adults. J Am Geriatr Soc (2015) 63(11):2227–46. 10.1111/jgs.13702 26446832

[12] Olfson M King M Schoenbaum M. Benzodiazepine use in the United States. JAMA Psychiatry (2015) 72(2):136–42. 10.1001/jamapsychiatry.2014.1763 25517224

[13] Chong Y Fryer CD Gu Q Prescription sleep aid use among adults: United States, 2005–2010. NCHS Data Brief (2013) 127:1–8. 24152538

[14] Salahudeen MS Duffull SB Nishtala PS. Anticholinergic burden quantified by anticholinergic risk scales and adverse outcomes in older people: a systematic review. BMC Geriatr (2015) 15:31. 10.1186/s12877-015-0029-9 25879993 PMC4377853

[15] Tune LE. Anticholinergic effects of medication in elderly patients. J Clin Psychiatry (2001) 62(Suppl 21):11–4. 11584981

[16] Maher RL Hanlon J Hajjar ER. Clinical consequences of polypharmacy in elderly. Expert Opin Drug Saf (2014) 13(1):57–65. 10.1517/14740338.2013.827660 24073682 PMC3864987

[17] Ziere G Dieleman JP Hofman A Pols HA van der Cammen TJ Stricker BH. Polypharmacy and falls in the middle age and elderly population. Br J Clin Pharmacol (2006) 61(2):218–23. 10.1111/j.1365-2125.2005.02543.x 16433876 PMC1885000

[18] Hanlon JT Schmader KE Samsa GP Weinberger M Uttech KM Lewis IK A method for assessing drug therapy appropriateness. J Clin Epidemiol (1992) 45(10):1045–51. 10.1016/0895-4356(92)90144-C 1474400

[19] Dalleur O Boland B Losseau C Henrard S Wouters D Speybroeck N Reduction of potentially inappropriate medications using the STOPP criteria in frail older inpatients: a randomised controlled study. Drugs Aging (2014) 31(4):291–8. 10.1007/s40266-014-0157-5 24566877

[20] Gallagher PF O’Connor MN O’Mahony D. Prevention of potentially inappropriate prescribing for elderly patients: a randomized controlled trial using STOPP/START criteria. Clin Pharmacol Ther (2011) 89(6):845–54. 10.1038/clpt.2011.44 21508941

[21] Hilmer SN Mager DE Simonsick EM Cao Y Ling SM Windham BG A drug burden index to define the functional burden of medications in older people. Arch Intern Med (2007) 167(8):781–7. 10.1001/archinte.167.8.781 17452540

[22] American Pharmacists Association, National Association of Chain Drug Stores Foundation. Medication therapy management in pharmacy practice: core elements of an MTM service model (version 2.0). J Am Pharm Assoc (2008) 48(3):341–53. 10.1331/JAPhA.2008.08514 18595820

[23] Chen Y Zhu LL Zhou Q. Effects of drug pharmacokinetic/pharmacodynamic properties, characteristics of medication use, and relevant pharmacological interventions on fall risk in elderly patients. Ther Clin Risk Manag (2014) 10:437–48. 10.2147/TCRM.S63756 24966681 PMC4063859

[24] Rowe JW Andres R Tobin JD Norris AH Shock NW The effect of age on creatinine clearance in men: a cross-sectional and longitudinal study. J Gerontol (1976) 31(2):155–63. 10.1093/geronj/31.2.155 1249404

[25] Tan JL Eastment JG Poudel A Hubbard RE. Age-related changes in hepatic function: an update on implications for drug therapy. Drugs Aging (2015) 32(12):999–1008. 10.1007/s40266-015-0318-1 26547855

[26] Wilhelm SM Kale-Pradhan PB. Estimating creatinine clearance: a meta-analysis. Pharmacotherapy (2011) 31(7):658–64. 10.1592/phco.31.7.658 21923452

[27] Panel on Prevention of Falls in Older Persons, American Geriatrics Society and British Geriatrics Society. Summary of the Updated American Geriatrics Society/British Geriatrics Society clinical practice guideline for prevention of falls in older persons. J Am Geriatr Soc (2011) 59(1):148–57. 10.1111/j.1532-5415.2010.03234.x 21226685

[28] Stevens JA Phelan EA. Development of STEADI: a fall prevention resource for health care providers. Health Promot Pract (2013) 14(5):706–14. 10.1177/1524839912463576 23159993 PMC4707651

[29] Tarn DM Paterniti DA Kravitz RL Fein S Wenger NS. How do physicians conduct medication reviews? J Gen Intern Med (2009) 24(12):1296–302. 10.1007/s11606-009-1132-4 19813063 PMC2787945

[30] Karapinar-Carkit F Borgsteede SD Zoer J Smit HJ Egberts AC van den Bemt PM Effect of medication reconciliation with and without patient counseling on the number of pharmaceutical interventions among patients discharged from the hospital. Ann Pharmacother (2009) 43(6):1001–10. 10.1345/aph.1L597 19491320

[31] JCoP Practitioners. Pharmacists’ Patient Care Process. (2014). Available from: http://www.pharmacist.com/sites/default/files/files/PatientCareProcess.pdf

[32] Petterson SM Liaw WR Phillips RL Rabin DL Meyers DS Bazemore AW. Projecting US primary care physician workforce needs: 2010–2025. Ann Fam Med (2012) 10(6):503–9. 10.1370/afm.1431 23149526 PMC3495923

[33] Jaen CR Stange KC Nutting PA. Competing demands of primary care: a model for the delivery of clinical preventive services. J Fam Pract (1994) 38(2):166–71. 8308509

[34] Gillespie LD Robertson MC Gillespie WJ Sherrington C Gates S Clemson LM Interventions for preventing falls in older people living in the community. Cochrane Database Syst Rev (2012) 9:CD007146 10.1002/14651858.CD007146.pub3 PMC8095069 22972103

[35] Pit SW Byles JE Henry DA Holt L Hansen V Bowman DA Quality Use of Medicines program for general practitioners and older people: a cluster randomised controlled trial. Med J Aust (2007) 187(1):23–30. 17605699 10.5694/j.1326-5377.2007.tb01110.x

[36] Hirsch JD Steers N Adler DS Kuo GM Morello CM Lang M Primary care-based, pharmacist-physician collaborative medication-therapy management of hypertension: a randomized, pragmatic trial. Clin Ther (2014) 36(9):1244–54. 10.1016/j.clinthera.2014.06.030 25085406 PMC4169745

[37] Planas LG Crosby KM Mitchell KD Farmer KC Evaluation of a hypertension medication therapy management program in patients with diabetes. J Am Pharm Assoc (2009) 49(2):164–70. 10.1331/JAPhA.2009.08164 19289342

[38] Chisholm-Burns MA Kim Lee J Spivey CA Slack M Herrier RN Hall-Lipsy E US pharmacists’ effect as team members on patient care: systematic review and meta-analyses. Med Care (2010) 48(10):923–33. 10.1097/MLR.0b013e3181e57962 20720510

[39] Bartlett D Pang N Massey C Evans P. Pharmacist consultations: simplifying daily drug regimens and providing education on fall risk for older adults. Consult Pharm (2015) 30(3):141–52. 10.4140/TCP.n.2015.141 25760664

[40] Modig S Holmdahl L Bondesson A. Medication reviews in primary care in Sweden: importance of clinical pharmacists’ recommendations on drug-related problems. Int J Clin Pharm (2016) 38(1):41–5. 10.1007/s11096-015-0189-x 26582483

[41] Mott DA Martin B Breslow R Michaels B Kirchner J Mahoney J Impact of a medication therapy management intervention targeting medications associated with falling: results of a pilot study. J Am Pharm Assoc (2016) 56(1):22–8. 10.1016/j.japh.2015.11.001 26802916 PMC4743551

[42] Cooper JW Burfield AH Medication interventions for fall prevention in the older adult. J Am Pharm Assoc (2009) 49(3):e70–82. 10.1331/JAPhA.2009.09044 19443314

